# Metagenomic Surveillance of Viral Gastroenteritis in a Public Health Setting

**DOI:** 10.1128/spectrum.05022-22

**Published:** 2023-07-11

**Authors:** Dennis Schmitz, Florian Zwagemaker, Bas van der Veer, Harry Vennema, Jeroen F. J. Laros, Marion P. G. Koopmans, Miranda De Graaf, Annelies Kroneman

**Affiliations:** a National Institute of Public Health and the Environment, Center for Infectious Disease Control, Bilthoven, The Netherlands; b Erasmus Medical Center, Viroscience, Rotterdam, The Netherlands; c Leiden University Medical Center, Department of Human Genetics, Leiden, The Netherlands; U.S. Food and Drug Administration

**Keywords:** next-generation sequencing, norovirus, surveillance, gastroenteritis, enteric viruses, public health, surveillance studies, transmissible gastroenteritis virus

## Abstract

Norovirus is the primary cause of viral gastroenteritis (GE). To investigate norovirus epidemiology, there is a need for whole-genome sequencing and reference sets consisting of complete genomes. To investigate the potential of shotgun metagenomic sequencing on the Illumina platform for whole-genome sequencing, 71 reverse transcriptase quantitative PCR (RT-qPCR) norovirus positive-feces (threshold cycle [*C_T_*], <30) samples from norovirus surveillance within The Netherlands were subjected to metagenomic sequencing. Data were analyzed through an in-house next-generation sequencing (NGS) analysis workflow. Additionally, we assessed the potential of metagenomic sequencing for the surveillance of off-target viruses that are of importance for public health, e.g., sapovirus, rotavirus A, enterovirus, parechovirus, aichivirus, adenovirus, and bocaparvovirus. A total of 60 complete and 10 partial norovirus genomes were generated, representing 7 genogroup I capsid genotypes and 12 genogroup II capsid genotypes. In addition to the norovirus genomes, the metagenomic approach yielded partial or complete genomes of other viruses for 39% of samples from children and 6.7% of samples from adults, including adenovirus 41 (*N* = 1); aichivirus 1 (*N* = 1); coxsackievirus A2 (*N* = 2), A4 (*N* = 2), A5 (*N* = 1), and A16 (*N* = 1); bocaparvovirus 1 (*N* = 1) and 3 (*N* = 1); human parechovirus 1 (*N* = 2) and 3 (*N* = 1); Rotavirus A (*N* = 1); and a sapovirus GI.7 (*N* = 1). The sapovirus GI.7 was initially not detected through RT-qPCR and warranted an update of the primer and probe set. Metagenomic sequencing on the Illumina platform robustly determines complete norovirus genomes and may be used to broaden gastroenteritis surveillance by capturing off-target enteric viruses.

**IMPORTANCE** Viral gastroenteritis results in significant morbidity and mortality in vulnerable individuals and is primarily caused by norovirus. To investigate norovirus epidemiology, there is a need for whole-genome sequencing and reference sets consisting of full genomes. Using surveillance samples sent to the Dutch National Institute for Public Health and the Environment (RIVM), we compared metagenomics against conventional techniques, such as RT-qPCR and Sanger-sequencing, with norovirus as the target pathogen. We determined that metagenomics is a robust method to generate complete norovirus genomes, in parallel to many off-target pathogenic enteric virus genomes, thereby broadening our surveillance efforts. Moreover, we detected a sapovirus that was not detected by our validated gastroenteritis RT-qPCR panel, which exemplifies the strength of metagenomics. Our study shows that metagenomics can be used for public health gastroenteritis surveillance, the generation of reference-sets for molecular epidemiology, and how it compares to current surveillance strategies.

## INTRODUCTION

Norovirus is the most common cause of viral gastroenteritis (GE) and results in significant morbidity and mortality in the young, elderly, and immunocompromised worldwide (1–6). To reduce the burden of norovirus disease, vaccines are being developed, and the first ones have completed phase II trials, but none have been approved yet (7, 8). Similarly, several drugs and biologicals have been tested off-label, such as ribavirin, immunoglobulins, and nitazoxanide, but conclusive evidence of their effectiveness is lacking (9, 10). These studies are complicated, as treatment and vaccine efficacy may in part be strain or genotype dependent.

Norovirus is a highly diverse genus and belongs to the *Caliciviridae* family. The *Norovirus* genus is currently divided in 10 genogroups (G), of which viruses belonging to GI, GII, GIV, GVIII, and GIX infect humans. The GI and GII genogroups are further subdivided into at least 48 genotypes (11). Of these, GII genotype 4 (GII.4) is the most prevalent, and due to antigenic drift, new GII.4 variants emerge which can become dominant and replace the previous variant (11). Diversity is further increased by recombination, with the open reading frame 1/2 (ORF1/2) junction as a recombination hot spot. Recombination usually occurs within a genogroup, but some intergenogroup recombinant genomes have also been identified (11–13). Therefore, for comprehensive genomic characterization of noroviruses, both ORF1 and ORF2 are typed.

Although norovirus surveillance to the genotype level is limited, the most common method to genotype norovirus in routine public health and food safety settings is by Sanger sequencing of the 3′ ORF1 end of the RNA-dependent RNA polymerase (RdRp; non-structural protein 7, NS7), the 5′ ORF2 end of the capsid (viral protein 1, VP1) coding domain regions (CDR), or a larger fragment containing both fragments and spanning the ORF1/2 junction. A classification scheme was developed in which the VP1 genomic sequence and the RdRp sequences are assigned to genogroups and genotypes to capture both antigenic divergence and the presence of recombinants (11, 14). The inferred genotypes and sequences support outbreak investigations and epidemiological surveillance of norovirus circulation (11, 14–16). While these partial genome sequences are informative, the generated sequences are generally too short to conclusively determine transmission chains within an outbreak.

With next-generation sequencing (NGS) techniques becoming more widely available, their application for sewage-based community surveillance and foodborne outbreak surveillance is being explored. NGS of difficult food and environmental sample matrices, which usually only contain low levels of viruses, frequently results in short sequences outside the canonical typing region. The identification and typing of these sequences would require a continuously updated reference set containing the complete genomic information of norovirus strains present in the human population. Full or even partial genomes generated by NGS would also provide a higher genetic resolution to investigate outbreaks and aid in genotype to phenotype studies. In the latest norovirus nomenclature update, the authors anticipated that the next update would be based on complete genome reference sequences, enabling typing of sequences outside the typing regions (11).

NGS generates reads from all taxa present in the sample, and with a metagenomic-focused analysis, reads are reconstituted to (partial) genomes, which are taxonomically classified based on their similarity to known strains in public databases. Therefore, in addition to targeted norovirus surveillance, metagenomics also provides an opportunity to detect several other viruses that cause GE in humans and are, therefore, relevant for public health, including Rotavirus A (RVA), enteroviruses (EV), and a range of other enteric viruses that are less frequent causes of outbreaks (17–21). Currently, a typical diagnostic approach is to use a multiplex reverse transcriptase quantitative PCR (RT-qPCR) to test for the presence of a range of GE viruses. Further characterization through targeted Sanger sequencing has been limited and is usually targeted to a single virus due to technical limitations and cost. However, with metagenomics, other viruses can be detected during norovirus-targeted NGS-based surveillance.

The primary goal of this study is to determine the robustness of a nontargeted NGS approach as a method to determine full norovirus genomes. Secondarily, we aim to assess its potential to provide off-target molecular characterization of GE pathogens that are relevant from a public health perspective. For this study we selected norovirus RT-qPCR-positive samples that were sent to the Dutch norovirus surveillance program by regional laboratories for referral in outbreak situations.

## RESULTS

### Complete norovirus genomes were robustly generated using metagenomics.

**FIG 1:**
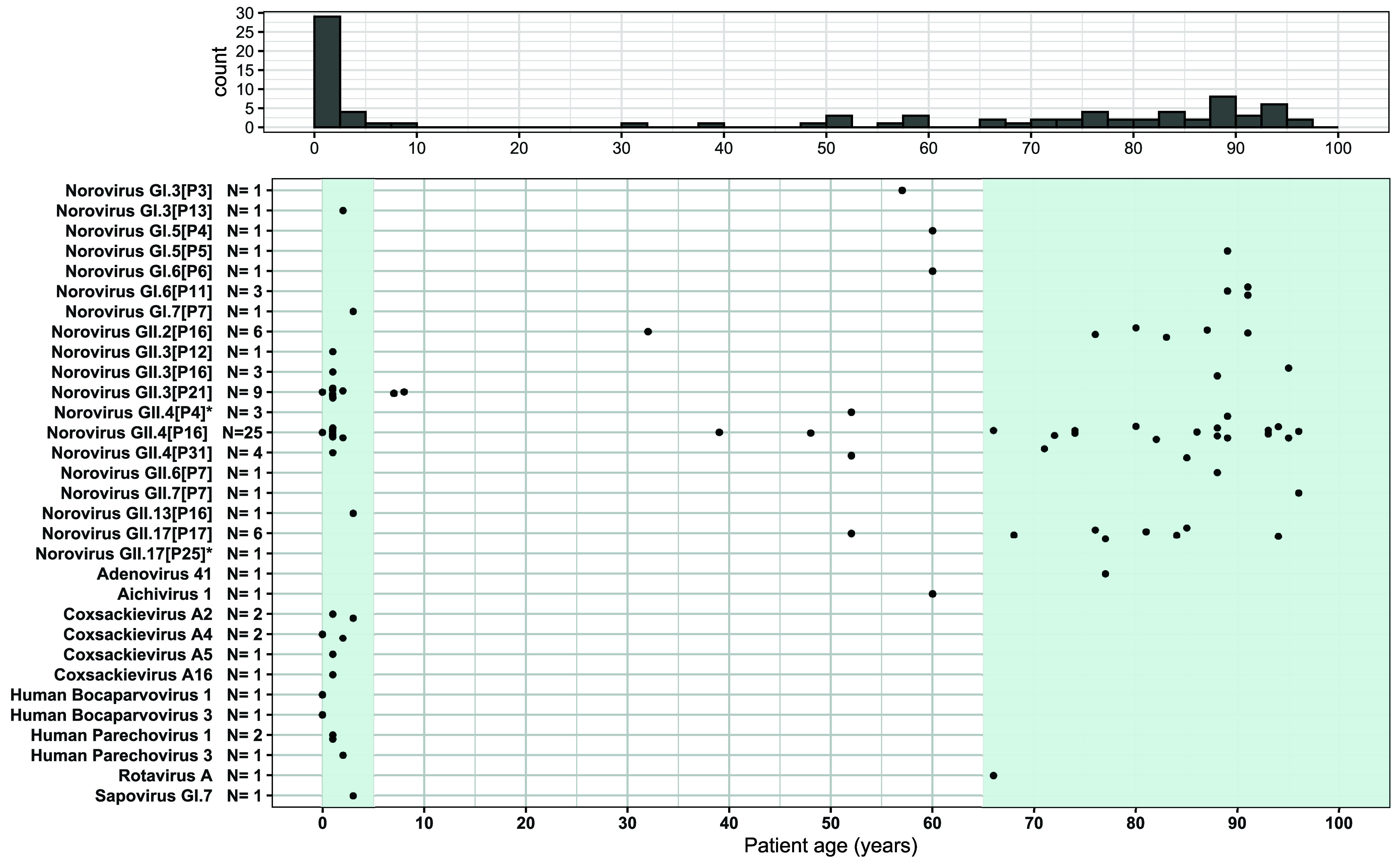
Patient age per detected virus species or norovirus genotype. The left light-gray rectangle shows young children (<5 years old), and the right one shows the elderly (>65 years old). The histogram depicts the total number of detected viruses of public health relevance per age group, with bins of 2.5 years. *, Norovirus GII.17[P25] (*N* = 1) and GII.4[P4] (*N* = 1) were present in the same sample and could not be plotted due to unknown patient age. *N*, total number of times a virus genome was observed.

To investigate the potential of shotgun metagenomic sequencing on the Illumina platform, 71 RT-qPCR norovirus-positive feces (threshold cycle [*C_T_*], <30) samples from norovirus surveillance within The Netherlands were subjected to metagenomics. Of the 71 samples, 66 (93%) yielded norovirus genomic sequences, while off-target viruses were identified in 13 (18%) of the samples (Fig. 1; Table 1). Of the 66 samples resulting in norovirus sequences, 58 (82%) yielded a full norovirus genome, and eight (11%) yielded a partial norovirus genome. Two full and two partial minority norovirus strains were additionally generated from double-infection samples, resulting in a total of 60 full and 10 partial norovirus sequences (Table 1). The five (7.9%) remaining samples were possibly negative due to either a low viral input, the presence of inhibitors, or the presence of another highly abundant taxon. Samples R02-07 and R02-09 had high viral loads (*C_T_*, <25) but contained a highly abundant adenovirus (AdV) (*C_T_*, 7.1; Table 1) and a 64-kb Pseudomonas phage with a 723× depth of coverage, respectively.

**TABLE 1:** Sample overview of 71 norovirus (NoV) RT-qPCR-positive samples^*a*^

Sample	Collection date (yr-mo-day)	Cluster no. (SNP distance)	NoV *C_T_* value^*b*^		Sanger NoV genotype^*c*^	WGS NoV genotype^*c*^	WGS major NoV strain		WGS minor NoV strain Length [bp (%)]; genotype^*c*^; avg. depth [×(SD)]	Off-target GE virus *C_T_* value^*c*^^,^^*d*^					WGS off-target GE virus length (bp [%])
			GI	GII			Length [bp (%)]	Avg. depth [×(SD)]		RVA	AdV	SaV	EV	HPeV	
R01-01	2016-01-25			11.7	GII.4[P4]	GII.4[P4]	7,561 (100)	10,849 (5790)							
R01-02	2016-04-08			13.9		GII.7[P7]	7,548 (100)	10,789 (3759)							
R01-03	2016-05-12		17.3		GI.3[P13]	GI.3[P13]	7,780 (100)	5,906 (2474)		31.8			33.9	28.8	HPeV3 (7,334 [78])
R01-04	2016-06			19.6	GII.17[P25]	GII.17[P25]	7,530 (100)	25 (10)	7,560 (55); GII.4[P4]; 3 (2)						
R01-05	2016-02-16		23.8		GI.5[P5]	GI.5[P5]	7,688 (100)	12,281 (2886)							
R01-06	2016-08-16		24.7			GI.7[P7]	7,735 (100)	186 (62)					^*e*^	^*e*^	SaV GI.7 (7,455 [100]);CV-A2 (7,352 [98])
R01-07	2016-03-01			27.1	GII.3[P21]	GII.3[P21]	7,552 (100)	214 (85)					34.0	30.9	HPeV1 (7,340 [100])
R01-20	2016-01-13		17.9		GI.3[P3]	GI.3[P3]	7,745 (100)	6,382 (2434)							
R02-06	2015-01-31			17.6	GII.17[P17]	GII.17[P17]	7,572 (100)	433 (298)							
R02-07	2015-02-26			20.6	GII.17[P17]						7.1				AdV41 (34,184 [100])
R02-08	2015-05-15	1 (0)		20.6	GII.17[P17]	GII.17[P17]	7,561 (100)	58 (30)							
R02-09	2015-05-27			16.8	GII.17[P17]										
R02-10	2015-05-15	1 (0)		20.8	GII.17[P17]	GII.17[P17]	7,561 (100)	285 (140)							
R02-11	2015-11-20			17.4	GII.17[P17]	GII.17[P17]	7,561 (100)	6,274 (2445)							
R02-12	2015-11-27			20.8	GII.17[P17]	GII.17[P17]	7,573 (100)	59 (34)							
R02-13	2016-03-30			20.7	GII.17[P17]	GII.17[P17]	7,560 (100)	1,588 (577)	7,572 (100); GII.4[P31]; 61 (231)						
R02-14	2016-03-30			22.5	GII.4[P4]	GII.4[P4]	7,560 (100)	508 (243)							
R02-15	2016-10		19.0		GI.6[P11]	GI.6[P11]	7,685 (100)	376 (306)							
R02-16	2016-10		17.0		GI.6[P11]	GI.6[P11]	7,688 (100)	3,830 (1734)							
R02-17	2016-11-28		19.8		GI.6[P11]	GI.6[P11]	7,701 (100)	124 (65)							
R02-18	2017-02-16	2 (0)	33.2	23.9	GII.2[P16]	GII.2[P16]	7,540 (93)	9 (6)							
R02-19	2017-02-16	2 (0)		18.9	GII.2[P16]	GII.2[P16]	7,552 (100)	1,397 (560)							
R02-20	2017-12-27			25.6	GII.6[P7]	GII.6[P7]	7,550 (23)	2(3)							
R02-21	2018-01-11	3 (1)		16.2	GII.2[P16]	GII.2[P16]	7,536 (100)	1,800 (780)							
R02-22	2018-01-12	3 (1)		21.5	GII.2[P16]	GII.2[P16]	7,536 (100)	41 (15)							
R02-23	2018-08-01			19.6	GII.2[P16]	GII.2[P16]	7,539 (100)	309 (120)							
R02-24	2018-07-03			22.0	GII.2[P16]	GII.2[P16]	7,605 (100)	1,143 (349)							
R03-01	2018-02-14			21.2		GII.4[P16]	7,555 (60)	5 (5)							
R03-02	2018-03-09		31.5	17.8		GII.4[P31]	7,560 (100)	586 (247)			36.0				
R03-03	2018-03-21			19.9		GII.4[P31]	7,563 (84)	10 (8)							
R03-04	2018-07-28			18.3	GII.4[P16]	GII.4[P16]	7,574 (100)	1,727 (639)							
R03-05	2018-08-22			21.1	GII.4[P16]	GII.4[P16]	7,564 (99)	35 (16)							
R03-06	2018-09-18			16.4	GII.4[P16]	GII.4[P16]	7,569 (100)	8,543 (3017)		31.0					
R03-07	2018-09-18			15.9	GII.4[P16]	GII.4[P16]	7,569 (100)	9,561 (3912)							
R03-08	2018-09-28			17.3	GII.4[P16]	GII.4[P16]	7,569 (100)	262 (97)							
R03-09	2018-10-03			20.9	GII.13[P16]	GII.13[P16]	7,497 (100)	168 (64)						27.86	
R03-10	2018-10-05			17.2	GII.4[P16]	GII.4[P16]	7,564 (100)	358 (167)							
R03-11	2018-08-16			18.7		GII.3[P16]	7,573 (100)	103 (44)							
R03-12	2018-10-19			18.1	GII.3[P16]	GII.3[P16]	7,543 (100)	116 (49)							
R03-13	2018-10-22			15.8	GII.4[P16]	GII.4[P16]	7,584 (99)	222 (76)							
R03-14	2018-11-10			20.8	GII.4[P16]	GII.4[P16]	7,569 (100)	224 (91)							
R03-15	2018-11-13			16.6		GII.4[P16]	7,568 (100)	1,093 (628)							
R03-16	2018-11-20			20.6	GII.4[P16]	GII.4[P16]	7,529 (100)	31 (15)	7,548 (65); GII.3[P21]; 5 (6)						
R03-17	2018-11-23			15.8	GII.4[P16]	GII.4[P16]	7,569 (100)	417 (156)							
R03-18	2018-11-29			18.5	GII.4[P16]	GII.4[P16]	7,569 (100)	1,574 (767)							
R03-20	2018-12-03			16.9	GII.4[P16]	GII.4[P16]	7,569 (100)	1,070 (336)							
R03-21	2018-12-07	4 (0)		16.1	GII.4[P16]	GII.4[P16]	7,563 (100)	50,155 (12,983)							
R03-22	2018-12-07	4 (0)		25.3	GII.4[P16]	GII.4[P16]	7,555 (95)	11 (7)							
R03-23	2018-12-10			18.0	GII.4[P16]	GII.4[P16]	7,569 (100)	4,126 (723)							
R03-24	2018-12-02	5 (1)		23.5	GII.3[P21]	GII.3P21]	7,546 (100)	150 (54)							
R04-01	2018-09-11			13.9	GII.3[P16]	GII.3[P16]	7,552 (100)	313 (129)		32.1	28.3		23.3		EV-A subspecies^*g*^
R04-02	2018-12-07			13.6	GII.4[P16]	GII.4[P16]	7,569 (99)	37 (13)							
R04-03	2018-11-03			14.6	GII.3[P12]	GII.3[P12]	7,566 (100)	10,467 (2942)					27.8	26.3	HPeV1 (7,344 [99])
R04-04	2019-01-03			20.5		GII.4[P16]	7,567 (99)	45 (16)							
R04-05	2018-08-30			22.3	GII.4[P16]	GII.4[P16]	7,569 (100)	531 (186)			33.0				
R04-06	2018-09-04			23.3	GII.4[P16]	GII.4[P16]	7,564 (86)	8 (5)			27.3				
R04-07	2017-12-04			24.8	GII.3[P21]	GII.3[P21]	7,543 (99)	77 (22)							
R04-08	2018-10-31	6 (2)		22.8	GII.3[P21]	GII.3[P21]	7,553 (100)	1,654 (705)							
R04-09	2018-10-31	6 (2)		19.7	GII.3[P21]	GII.3[P21]	7,553 (100)	2,000 (879)					25.8		CV-A16 (7,363 [91])
R04-10	2018-11-30			24.8	GII.4[P16]	GII.4[P16]	7,578 (44)	3 (2)		10.6					RVA^*f*^
R04-11	2018-12-05			20.5	GII.4[P31]	GII.4[P31]	7,563 (100)	8,353 (2742)							
R04-12	2018-12-10			18.8	GII.4[P16]	GII.4[P16]	7,563 (100)	5,926 (1419)					27.1		CV-A5 (7,404 [100])
R04-13	2018-12-10			23.3	GII.3[P21]	GII.3[P21]	7,544 (96)	13 (6)			23.9				
R04-14	2018-12-21			18.9	GII.4[P16]	GII.4[P16]	7,570 (100)	43,377 (12,610)				33.0	25.9	33.6	HBoV1 (5,491 [99]); HBoV3 (5,432 [100]); CV-A4 (7,437 [100])
R04-15	2018-12-12		22.1		GI.5[P4]	GI.5[P4]	7,700 (100)	148 (45)	7,688 (99); GI.6[P6]); 19 (22)						AiV-1 (8,252 [100])
R04-16	2018-09-27			29.3											
R04-17	2018-10-09			25.1	GII.3[P21]	GII.3[P21]	7,536 (99)	42 (17)			27.4				
R04-18	2018-11-29			21.7	GII.4[P16]	GII.4[P16]	7,565 (100)	343 (87)					27.8		CV-A2 (7,357 [100])
R04-20	2018-12-13			29.0									22.1		CV-A4 (7,427 [100])
R04-21	2019-03-03			24.3											
R04-22	2018-12-05	5 (1)		19.4		GII.3[P21]	7,549 (100)	212 (120)							

a RT-qPCR and Sanger sequencing results are shown alongside metagenomic results for NoV and several off-target relevant viruses.

b Blank cells represent samples that tested negative.

c All NoV GII.4 (ORF2/capsid) genotyped sequences were from the Sydney 2012 variant, and all NoV.P4 (ORF1/RdRp) genotyped sequences were from the New Orleans 2009 variant.

d RVA, Rotavirus A; AdV, adenovirus; SaV, sapovirus; EV, enterovirus; HPeV, human parechovirus; CV, coxsackievirus; HBoV, human bocaparvovirus; AiV, aichivirus.

e Not tested due to sample depletion.

f All 11 segments (genotype, length, BoC at a depth of coverage of ≥3): segment 1 (R1, 3,302, 100%), segment 2 (C1, 2,729, 100%), segment 3 (M1, 2,591, 100%), segment 4 (P8, 2,359, 100%), segment 5 (A1, 1,566, 100%), segment 6 (I1, 1,356, 100%), segment 7 (T1, 1,032, 100%), segment 8 (N1, 1,059, 100%), segment 9 (G9, 1,061, 100%), segment 10 (E1, 750, 100%), segment 11 (H1, 664, 100%).

g Contained several *Enterovirus A* contigs that could not be reconstituted into a single taxon.

**FIG 2:**
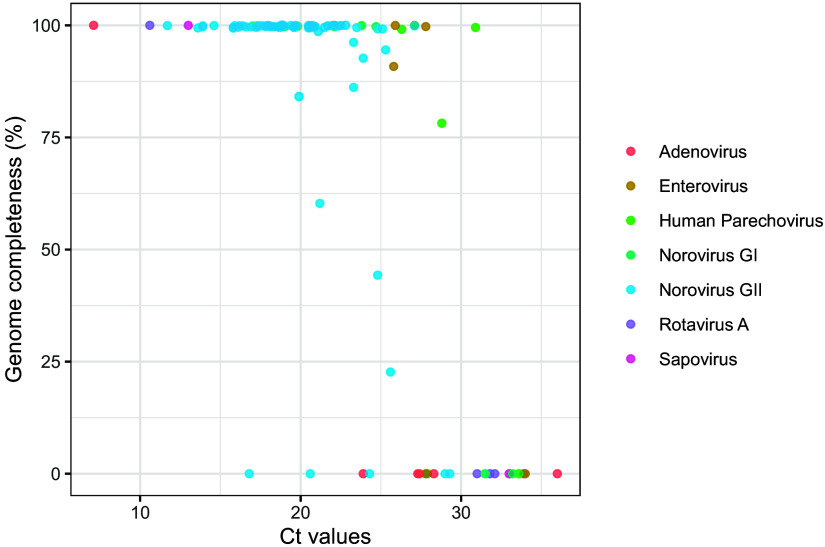
Percentage of genome completeness of norovirus and off-target GE viruses versus *C_T_*-values. Genome completeness was determined for a minimum 3× depth of coverage.

**FIG 3:**
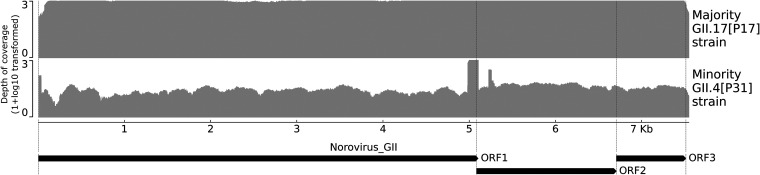
Example depth of coverage plot of norovirus (NoV) GII strains in sample R02-13. Two strains were present in this sample, a GII.17[P17] norovirus strain (top) with high depth of coverage (DoC) and a GII.4[P31] norovirus strain (bottom) with lower DoC, denoted “major” and “minor,” respectively. The characteristic block-like high coverage peaks near the ORF1/2 junction of the minority strain are due to homology between the strains in these regions. The aligned reads of the majority strain adversely affect the consensus sequence of the minority strain, necessitating manual correction. A full overview of all norovirus GI, GII, and off-target viruses with public health relevance is shown in Fig. S1 to S4. The coverage is shown as a 1 + log_10_ transformation with a maximum 999× depth of coverage.

A comparison of RT-qPCR values versus genome completeness at a sequencing depth cutoff of 3 showed that above a *C_T_* value of 27.1, no norovirus contigs were obtained (Fig. 2). For the mixed-strain samples, block-like coverage peaks near the ORF1/2 junction were observed as a result of homologous regions between these strains, and these regions had to be resolved manually (Fig. 3).

### Metagenomic sequencing of norovirus has higher sensitivity than Sanger sequencing.

For 60 out of 71 samples, a Sanger norovirus sequence could be retrieved, compared to 66 for metagenomic sequences. To investigate the concordance and resolution of NGS versus Sanger sequencing, sequences generated by both methods were compared using a pairwise single nucleotide polymorphism (SNP) distance method with a pairwise deletion option that removes ambiguous positions. In total, 50 high-quality Sanger sequences were compared to NGS sequences. SNP differences were identified for six samples: R03-06, R03-07, R03-12, R03-13, and R04-07 had 1 SNP difference, while R03-09 had 2 SNP differences over a 969-nucleotide (nt) region (see Fig. S5 in the supplemental material). Although ambiguous positions were ignored by this method, NGS could clearly resolve ambiguous nucleotides that arose due to overlapping fluorescent peaks in the Sanger trace files.

### Phylogenetic analysis of norovirus strains.

**FIG 4:**
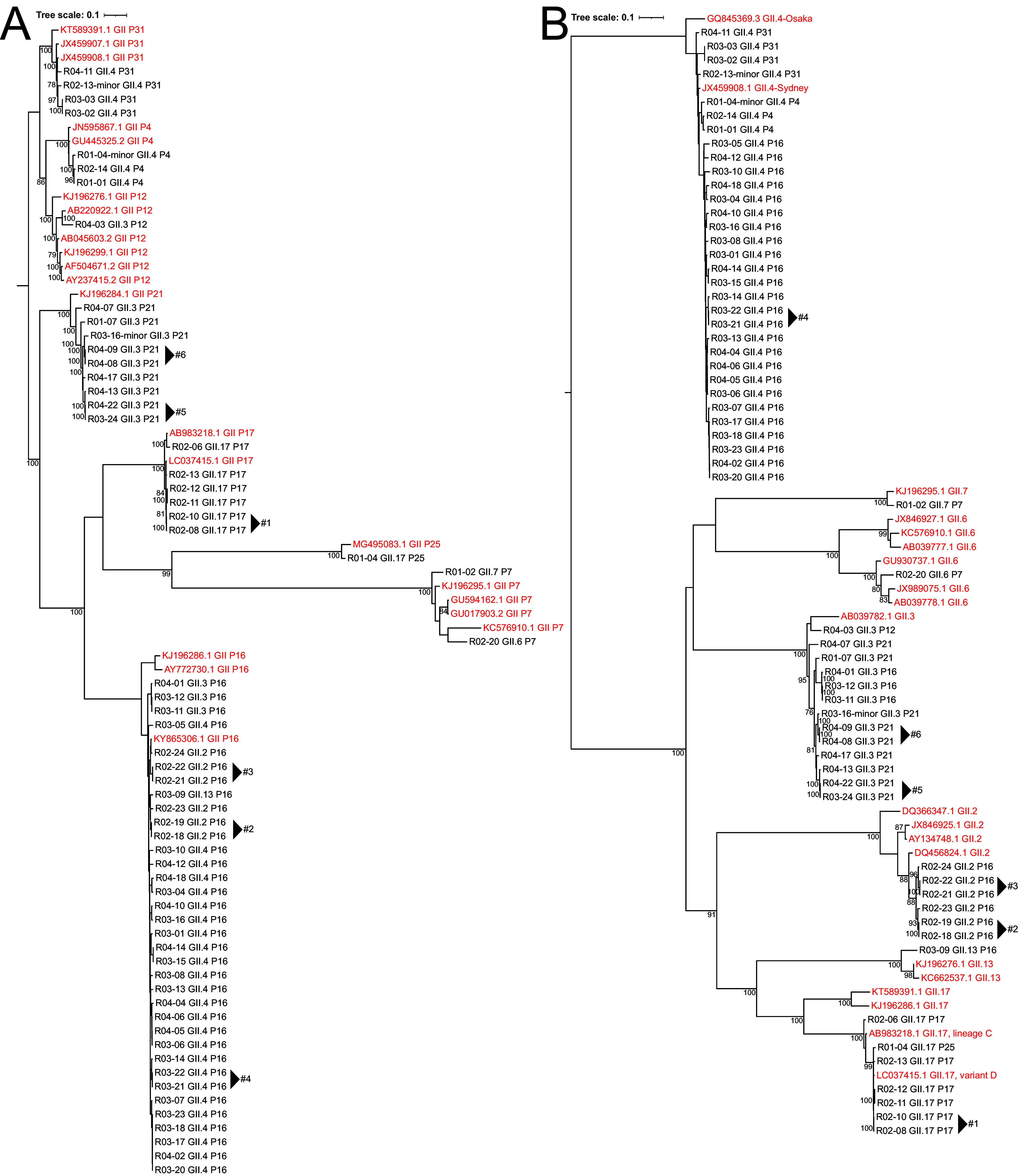
Maximum likelihood tree of 61 GII strains. (A) Maximum likelihood trees were inferred for ORF1 based on 5,166 nucleotides using the GTR best-fit model. (B) ORF2 based on 1,689 nucleotides using the TIM2 best-fit model. Reference strains from Chhabra et al. (11) are shown in red; clusters are annotated with numbered arrows (Table 1). The scale bar represents nucleotide substitutions per site, and selected bootstrap values greater than 70 are shown. For samples containing multiple strains, the lowest depth of coverage strain is denoted with “minor” in the tip label (e.g., “R01-04-minor”). All GII.4 and GII.P4 strains were Sydney and New Orleans variants, respectively.

**FIG 5:**
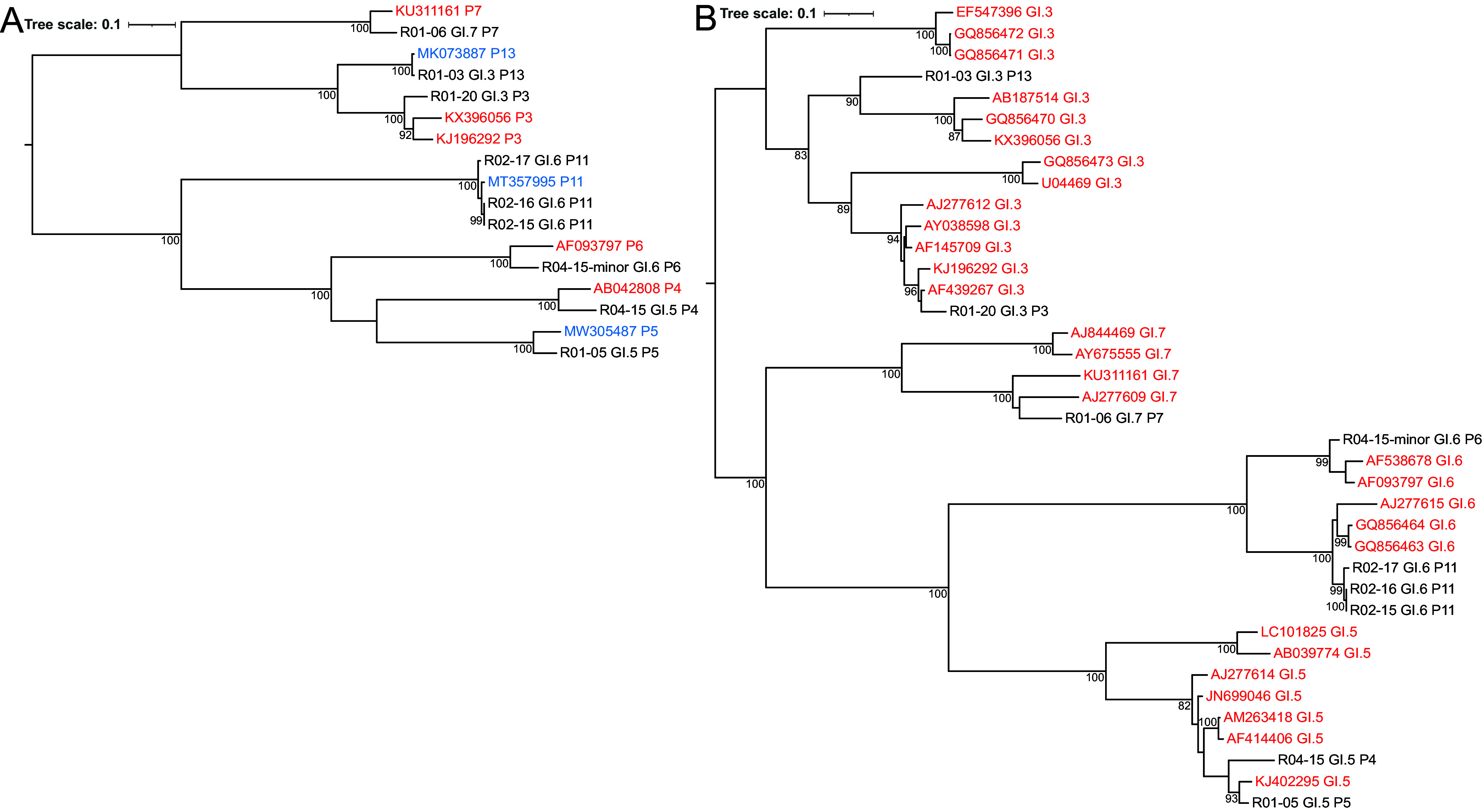
Maximum likelihood tree of 9 GI strains. (A and B) Maximum likelihood trees were inferred for (A) ORF1 based on 5,412 nucleotides using the TIM2 best-fit model and (B) ORF2 based on 1,665 nucleotides using the TIM2 best-fit model. Genotype reference strains from Chhabra et al. (11) are shown in red, while additional references from NCBI GenBank are shown in blue. The scale bar represents nucleotide substitutions per site, and selected bootstrap values greater than 70 are shown. In sample R04-15, two strains were detected, with the strain of lower coverage denoted as “minor” in the tip label.

To investigate the genetic diversity of the obtained norovirus sequences, we inferred maximum likelihood trees of the ORF1 and ORF2 sequences for norovirus GII (Fig. 4) and GI (Fig. 5). We included references from the latest nomenclature update described in Chhabra et al. (11). For samples that were linked in location and time (sampled within 1 week of each other), we investigated if there were differences in single nucleotide polymorphisms (SNPs). We identified six linked sample pairs, that had up to two SNP distances between them over the entire combined ORF1 to ORF3 coding domain sequence (CDS) (Table 1; Fig. 4).

GII.17[P25] strains are not often detected; therefore, we further investigated the origin of ORF2 and ORF1. For norovirus GII.17 strains, there are several lineages (A to D), of which variants C (GenBank accession number AB983218) and D (accession number LC037415) are contemporary strains that recently caused large outbreaks in Asia (22, 23). All GII.17 strains in this study belonged to variant D, with the exception of R02-06, which belonged to variant C (Fig. 4). The GII.17[P25] strain (R01-04) belonged to GII. 17 variant D (Fig. S6B), and it had a single amino acid change from the reference strain, which suggests a recent recombination event. Additionally, its polymerase region clustered with GII.P25 (Fig. S6A). This strain represents the second complete GII.P25 ORF1 sequence alongside MG495083.

### Children were frequently coinfected with another GE virus.

From the samples that tested positive for norovirus by RT-qPCR, 18% yielded off-target GE viruses, resulting in 13 full and two partial genomes (Table 1; Fig. 2 and 5). Children were more often coinfected with another GE virus (39%) than adults (6.7%; Fig. 1). Aichivirus 1 (AiV-1), RVA, and AdV41 were detected in individuals of 60, 66, and 71 years old, respectively, while all other off-target GE viruses were detected in samples from individuals younger than 5 years (Fig. 1). All detected EV were coxsackieviruses (CV). For several of the GE viruses, the number of sequences generated in this study provided a marked increase in the number of sequences uploaded to NCBI between 2017 and 2022 (Table S1).

To investigate the sensitivity of metagenomic sequencing for the off-target GE viruses, (RT-)qPCRs were performed for RVA, AdV, sapovirus (SaV), EV, and human parechovirus (HPeV) on 71 samples. For AiV and human bocaparvovirus (HBoV), (RT-)qPCR assays were not available (Table 1). The sample size was limited, but overall, no sequences were obtained with a *C_T_* value of >28 except for two HPeV genomes, while RT-qPCR detected several off-target viruses with a *C_T_* of >28, which were not detected with metagenomics, metagenomics allowed for the reliable detection and genotyping of off-target viruses in a single assay.

### Metagenomics identified a primer/probe mismatch for SaV GI.7.

An SaV GI.7 was initially identified by metagenomics but not by RT-qPCR. Based on the obtained SaV GI.7 sequence, the SaV RT-qPCR was updated (Table 1). The forward primer remained unchanged (5′-GAYCWGGCYCTCGCCACCT-3′), the probe was truncated from 5′-TGYACCACCTATRAACCAVGG-3′ to 5′-TGYACCACCTATRAACCA-3′, and the reverse primer was truncated at the 3′ end and extended on the 5′ end: 5′-GCCCTCCATYTCAAACACTAWTTTG-3′ to 5′-CATTGCCCTCCATYTCAAACACTA-3′. This updated primer set allowed for the retrospective detection of SaV with a *C_T_* of 13.

## DISCUSSION

As previously reported by others and shown in this study (24), Illumina-based metagenomic sequencing is a robust method for generating norovirus sequences. We were also able to determine norovirus genomes from minority strains in double-infection samples in which Sanger sequencing could not differentiate the minor from the major strain.

Although this study did not primarily investigate outbreaks, we identified several pairs of samples that were collected from the same location and time. These pairs had a pairwise SNP distance of ≤2 across the combined ORF1 to ORF3 CDS. However, to identify norovirus clusters, further investigation of outbreaks with well-characterized transmission chains is necessary to determine an appropriate SNP cutoff.

When comparing genome completeness against qPCR *C_T_* values, a proxy for the viral load, an overall lower genome completeness at higher *C_T_* values was observed, as reported by others (25). The genome ends had a lower coverage depth, but the reads were evenly distributed across the rest of the genome (26–29). For three samples with low *C_T_* value, no norovirus sequences could be obtained, and we hypothesize that this was caused by other abundant taxa outcompeting norovirus nucleic acids for sequencing space or the presence of inhibitors negatively affecting amplification efficiency (30). This limitation highlights one of the challenges of using agnostic, metagenomics-based virus typing.

In assessing the off-target coinfections, CV-A16 (B1a), CV-A2, CV-A4, and CV-A5 genomes were identified in infants. CV is the causative agent of hand, foot, and mouth disease (HFMD), which sporadically causes neurological symptoms, is linked to intrauterine fetal demise (31–33), and has recently caused large HFMD outbreaks in China (34–38). HPeV 1 and 3 were also identified in infants and can result in GE, and sporadically in neurological symptoms and death, in young children (39–43).

Moreover, an RVA coinfection, which causes severe GE (2), was identified in an elderly individual, and all 11 segments were genotyped. Importantly, this study was performed in The Netherlands, where RVA will be included in the National vaccination program in 2024. Metagenomic GE surveillance can help assess the effect of vaccination on the epidemiology of RVA in The Netherlands. The other off-target viruses that were detected were an AiV1, which is linked to predominantly subclinical GE (44, 45), AdV41, a known cause of GE (2), an HBoV1, and an HBoV3, the former of which is a respiratory virus, while the latter is a GE-causing virus (17, 19, 46). Lastly, an SaV GI.7 was identified in an infant, which belongs to the same viral family as norovirus with similar clinical and epidemiological characteristics (47, 48). These off-target findings provide a reference set to assess silent transmission and to facilitate genotype to phenotype studies to determine if genetic or genotype-specific factors contribute to severe pathogenesis. However, it should be noted that studies have shown that 7 to 15% of norovirus infections remains asymptomatic, meaning that some of these off-target viruses could be the causative agent of the reported gastroenteritis (49).

Interestingly, the SaV GI.7-positive sample that initially tested negative with RT-qPCR was identified by metagenomics. Evaluation of the primers and probe showed that these were not compatible with this genotype. Therefore, they were updated, and now they are similar to the frequently used primer and probe sets of Oka et al. (50). Since Okada et al. first detected SaV GI.7 in 2006, only a few GI.7 sequences have been submitted to NCBI, and the first complete genome was sequenced in 2018 (51, 52). The SaV GI.7 complete genome generated in this study most closely resembles this complete genome (GenBank accession number AB522390) with only 89% nucleotide identity, while SaV GI is shown to have low intragenotype diversity (51, 53). A recent large-scale review of SaV prevalence (48) did not report the GI.7 genotype in surveillance programs. Therefore, our results raise questions about where this genotype circulated without being detected in surveillance programs.

Even though this study had a limited sample size, it generated a marked addition of contemporary and publicly available full genomes. Compared to full-genome sequences uploaded to NCBI over a 4-year period, the genomes generated in this study represent up to a 33% increase in contemporary sequences for some of these off-target viruses (Table S1). This can help contextualize molecular epidemiology and outbreak tracing.

While Illumina metagenomic NGS is a more costly technique than RT-qPCR or Sanger sequencing, it has broad surveillance potential. Conventional methods require continuous updates of primers and probe sets to account for antigenic drift and novel genotypes. Without metagenomic-based surveillance, the SaV GI.7 in this study would not have been identified, and similar results are reported by others (54). Primer mismatches can also occur for viruses of chronically infected patients, where prolonged replication can result in mutations in the otherwise conserved domains targeted by Sanger sequencing assays.

In-depth analysis of NGS data to identify GE viruses in a public health setting is challenging and time-consuming. Negative controls and detection thresholds have to be optimized to account for lab-specific contamination levels to mitigate false-positives. Likewise, a mixed infection with shared homologous regions requires careful manual curation. Here, it helps to have standardized pipelines, with parameter settings tailored to the local laboratory methods and visualization of genomic regions for manual in-depth inspection.

In conclusion, this study shows the potential of NGS-based norovirus surveillance for generating a full-genome reference set for a broad range of public health-relevant pathogens and for high-resolution outbreak detection. By describing the caveats and strengths of metagenomic-based surveillance, and by sharing these complete genome sequences, we hope to help other researchers contextualize their outbreak investigations and improve molecular epidemiology.

## MATERIALS AND METHODS

### Sample selection.

A total of 71 norovirus RT-qPCR positive fecal samples obtained between 2015 and 2019 with a *C_T_* of <30 for at least one norovirus genogroup were selected from GE outbreak-related samples sent to the Dutch National Institute for Public Health and the Environment (RIVM) norovirus surveillance program by medical microbiology laboratories for referral. The samples originated predominantly from young children (<5 years old, *N* = 23) and elderly (>65 years old *N* = 38). For one sample, the patient age was not reported. *C_T_* values ranged from 17.0 to 33.2 (*N* = 10) and 11.7 to 29.3 (*N* = 63) for GI and GII, respectively. Feces samples were stored at 4°C until processing, which is the standard procedure at the RIVM for norovirus-positive feces.

### qPCR and Sanger sequencing.

Feces sample was suspended in 1,000 μL modified Eagle medium (MEM) medium containing gentamicin (Thermo Fisher, Bleiswijk, The Netherlands) to give a 10 to 20% vol/vol suspension and centrifuged at 16,100 relative centrifugal force (RCF) for 1 min at room temperature. Total nucleic acid (TNA) was extracted using the MagNA Pure 96 system (Roche, Almere, The Netherlands) and eluted in 50 μL elution buffer. All samples were subjected to a qPCR panel targeting norovirus GI, norovirus GII, RVA, sapovirus (SaV), and adenovirus (AdV). Norovirus Sanger sequences were generated as described previously (3, 15). Sanger sequences were analyzed using BioNumerics (AppliedMaths, Sint-Martens-Latem, Belgium) and genotyped via the NoroNet norovirus typing-tool (16). Additional viruses detected by NGS in this study, which are of relevance for public health, were confirmed by RT-qPCR: EV and human parechovirus (HPeV) via RT-qPCR as described by Benschop et al. (55, 56) and SaV via RT-qPCR with modified primers (as described above).

### Virus enrichment, NGS pretreatment, and NGS.

Feces was suspended in 1,000 μL Eagle MEM containing gentamicin (Thermo Fisher, Bleiswijk, The Netherlands) to give a 10 to 20% vol/vol suspension and centrifuged at 16,100 RCF for 1 min at room temperature. The supernatant was filtered using Costar Spin-X 0.45-μm CA membrane centrifuge tube filters (Corning, Amsterdam, The Netherlands). Afterward, 200 μL filtrate was supplemented with 25 μL 25 mM MgCl_2_ and treated with 1.25 μL 200 U/μL OmniCleave endonuclease (EpiCentre, Leusden, The Netherlands) for 1 h at 37°C.

Total nucleic acid (TNA) was extracted using the Magna Pure 96 system (Roche, Almere, The Netherlands) and eluted in 50 μL elution buffer. First-strand cDNA synthesis was performed using SuperScript III (Invitrogen, Bleiswijk, The Netherlands) using 11 μL TNA eluate as input. The second cDNA strand was synthesized using the NEBNext mRNA second-strand synthesis module (New England Biolabs, Leusden, The Netherlands) using 20 μL as input, as per the manufacturer’s instructions, resulting in 160 μL output. Next, the sample was purified using DNA Clean & Concentrator-5 capped columns (Zymo Research, Leiden, The Netherlands), as per the manufacturer’s instructions, resulting in 15 μL double-stranded DNA (dsDNA) per sample.

Library preparation was performed with the Nextera XT DNA library prep kit (Illumina, Eindhoven, The Netherlands). Samples were processed in four independent NGS runs, denoted with the prefixes R01 to R04. Prefixes R02, R03, and R04 were 150-nucleotide paired-end sequenced on an Illumina NextSeq instrument per the manufacturer’s specifications, and samples starting with R01 were 300-nucleotide paired-end sequenced on an Illumina MiSeq instrument by BaseClear B.V. (Leiden, The Netherlands). On average, 4.7 million reads (postfiltering) were generated per sample.

### Data analysis.

All NGS data were analyzed using an in-house NGS analysis workflow called Jovian (v1.01, https://github.com/DennisSchmitz/Jovian) using default settings. Briefly, this workflow removes human and poor-quality reads (i.e., nucleotides with a Phred score of <20 are trimmed, and reads with a length of <50 nt are discarded), assembles reads into contigs and annotates contigs of ≥250 nt via megaBLAST against the NCBI NT database, determines the lowest common ancestor (LCA) of the BLAST results, and finally, performs genotyping of several clinically important virus families and genera via their respective typing tools (16).

Norovirus sequences were manually curated and subsequently scaffolded and assembled based on same-genotype public reference sequences or by extending the 5′ and 3′ ends using soft-clipped overhangs. This manually assembled draft genome was then assessed and corrected by performing Minimap2 (57) alignment and LoFreq (58) SNP-calling, with manual curation of the genome ends, to produce a final sequence. Homologous regions of mixed-strain samples were manually corrected and curated. Any reference nucleotide with <3 reads coverage was masked with an N in the final genome sequence. Considering a minimum Phred score of 20 and minimum depth of coverage of 3, a sequencing error in the consensus requires ≥2/3 simultaneous errors which, by binomial distribution, has a 0.03% chance. This is a lower bound since the depth of coverage generally was ≥1 order of magnitude higher than 3 (Table 1).

Virus genomes were considered complete when their entire CDS was characterized with ≤100 N’s, and partial when it contained >100 N’s or could not be scaffolded to a full CDS sequence. If fewer than 50 total reads aligned against same-taxon scaffolds with a length ≥250 nt, they were considered negative. Norovirus scaffolds with a different ORF1 and ORF2 genotype than their majority strain were considered minority strains.

Norovirus sequences were aligned with reference strains from Chhabra et al. (11) using MUSCLE v3 (59). Maximum likelihood trees were inferred with IQ-TREE 2 (60), using 100 Felsenstein bootstraps (61) with best-fit models as identified by ModelFinder (62) and were edited with iTOL v5 (63). Results were visualized via ggplot2 and deeptools (64, 65).

### Data availability.

All filtered, General Data Protection Regulation-compliant, FASTQ files and full genomes were submitted to NCBI SRA under study accession number PRJEB54724 (accession numbers OP162334 to OP162342, OP205527 to OP205585 and OP255971 to OP255995). Sequences that contained too many N’s could not be uploaded to NCBI but are available on request. All complete and partial norovirus genomes were also uploaded to NoroNet (https://www.rivm.nl/en/noronet).
