# Supplementary material for: Metagenomic Surveillance of Viral Gastroenteritis in a Public Health Setting
Source: Microbiol Spectr. 2023 Jul 11;11(4):e05022-22. doi: 10.1128/spectrum.05022-22 (PMC10434279; doi:10.1128/spectrum.05022-22)
Supplement: Supplemental file 1 — Supplemental material. Download spectrum.05022-22-s0001.pdf, PDF file, 0.5 MB [file spectrum.05022-22-s0001.pdf]

Supplemental data

Supplemental Table S1 – NCBI nucleotide queries for GE viruses, uploaded between January 1st 2017 and January 1st 2022 compared to the sequences generated in this study.

Virus	Category	Query	No of sequences	No of sequences from this study
NoV	All sequences	"Norwalk virus"[porgn:__txid11983] AND ("2017/01/01"[PDAT] : "2022/01/01"[PDAT])	20116	
	Complete genome	"Norwalk virus"[porgn:__txid11983] AND ("2017/01/01"[PDAT] : "2022/01/01"[PDAT]) AND ("7200"[SLEN] : "10000"[SLEN])	1573	60 (3.8%)
RVA*	All sequences	"Rotavirus A"[porgn:__txid28875] AND ("2017/01/01"[PDAT] : "2022/01/01"[PDAT])	41440*	
	Complete CDS sequence	(("Rotavirus A"[porgn:__txid28875] AND ("2017/01/01"[PDAT] : "2022/01/01"[PDAT]))) AND "complete CDS"	19792*	11* (0.0%)
SaV	All sequences	("Sapovirus"[porgn:__txid95341] AND ("2017/01/01"[PDAT] : "2022/01/01"[PDAT]))	2163	
	Complete genome	(("Sapovirus"[porgn:__txid95341] AND ("2017/01/01"[PDAT] : "2022/01/01"[PDAT])) AND ("7000"[SLEN] : "10000"[SLEN]))	253	1 (5.0%)
AdV41	All sequences	("Human adenovirus 41"[porgn:__txid10524] AND ("2017/01/01"[PDAT] : "2022/01/01"[PDAT]))	546	
	Complete genome	("Human adenovirus 41"[porgn:__txid10524] AND ("2017/01/01"[PDAT] : "2022/01/01"[PDAT])) AND ("34000"[SLEN] : "40000"[SLEN])	20	1 (5.0%)
AiV1	All sequences	"Aichi virus 1"[porgn:__txid1313215] AND ("2017/01/01"[PDAT] : "2022/01/01"[PDAT])	245	
	Complete genomes	"Aichi virus 1"[porgn:__txid1313215] AND ("2017/01/01"[PDAT] : "2022/01/01"[PDAT])) AND ("8000"[SLEN] : "10000"[SLEN])	6	1 (16.6%)
CV-A2	All sequences	("Coxsackievirus A2"[porgn:__txid33757] AND ("2017/01/01"[PDAT] : "2022/01/01"[PDAT]))	307	
	Complete genome	("Coxsackievirus A2"[porgn:__txid33757] AND ("2017/01/01"[PDAT] : "2022/01/01"[PDAT])) AND ("7000"[SLEN] : "10000"[SLEN])	41	2 (4.8%)
CV-A4	All sequences	("Coxsackievirus A4"[porgn:__txid42785] AND ("2017/01/01"[PDAT] : "2022/01/01"[PDAT]))	790	
	Complete genome	("Coxsackievirus A4"[porgn:__txid42785] AND ("2017/01/01"[PDAT] : "2022/01/01"[PDAT])) AND ("7000"[SLEN] : "10000"[SLEN])	57	2 (3.5%)
CV-A5	All sequences	("Coxsackievirus A5"[porgn:__txid42786] AND ("2017/01/01"[PDAT] : "2022/01/01"[PDAT]))	219	
	Complete genome	("Coxsackievirus A5"[porgn:__txid42786] AND ("2017/01/01"[PDAT] : "2022/01/01"[PDAT])) AND ("7000"[SLEN] : "10000"[SLEN])	71	1 (1.4%)
CV-A16	All sequences	("Coxsackievirus A16"[porgn:__txid31704] AND ("2017/01/01"[PDAT] : "2022/01/01"[PDAT]))	3262	

	Complete genome	("Coxsackievirus A16"[porgn:__txid31704] AND ("2017/01/01"[PDAT] : "2022/01/01"[PDAT])) AND ("7000"[SLEN] : "10000"[SLEN])	133	1 (0.8%)
HPeV 1	All sequences	("Human parechovirus 1"[porgn:__txid12063] AND ("2017/01/01"[PDAT] : "2022/01/01"[PDAT]))	223	
	Complete genome	("Human parechovirus 1"[porgn:__txid12063] AND ("2017/01/01"[PDAT] : "2022/01/01"[PDAT])) AND ("7000"[SLEN] : "10000"[SLEN])	12	2 (16.7%)
HPeV 3	All sequences	("Human parechovirus 3"[porgn:__txid195055] AND ("2017/01/01"[PDAT] : "2022/01/01"[PDAT]))	386	
	Complete genome	("Human parechovirus 3"[porgn:__txid195055] AND ("2017/01/01"[PDAT] : "2022/01/01"[PDAT])) AND ("7000"[SLEN] : "10000"[SLEN])	57	1 (1.8%)
hBoV 1	All sequences	("Human bocavirus 1"[porgn:__txid689403] AND ("2017/01/01"[PDAT] : "2022/01/01"[PDAT]))	186	
	Complete genome	("Human bocavirus 1"[porgn:__txid689403] AND ("2017/01/01"[PDAT] : "2022/01/01"[PDAT])) AND ("4800"[SLEN] : "70000"[SLEN])	15	1 (6.7%)
hBoV 3	All sequences	("Human bocavirus 3"[porgn:__txid638313] AND ("2017/01/01"[PDAT] : "2022/01/01"[PDAT]))	73	
	Complete genome	("Human bocavirus 3"[porgn:__txid638313] AND ("2017/01/01"[PDAT] : "2022/01/01"[PDAT])) AND ("4800"[SLEN] : "70000"[SLEN])	3	1 (33.3%)

[*]Rotavirus A has 11 segments, each uploaded as their own database entry.

[#]Norovirus (NoV), rotavirus A (RVA), sapovirus (SaV), adenovirus (AdV), coxsackievirus (CV), human parechovirus (HPeV) and human bocavirus (hBoV).

[†] Enclosed in brackets is the percentage of sequences generated in this study, shown as a proportion of the total number of sequences uploaded to NCBI between 2017 and 2022.

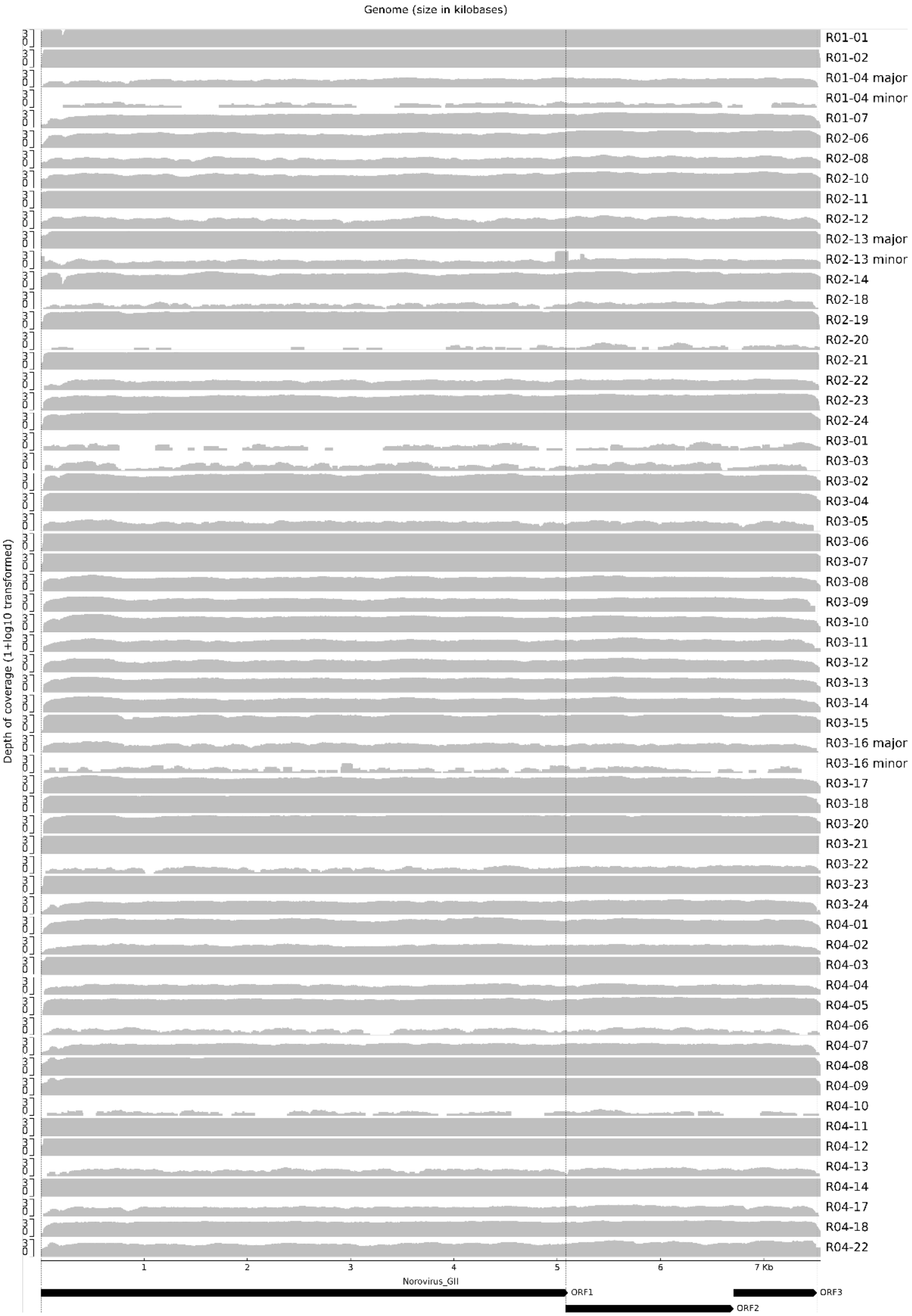

Supplemental Figure S1 – Sequence coverage plots of norovirus GII. Open Reading Frames (ORFs) are shown below the chart. The coverage is shown as a 1+log10 transformation with a maximum depth of 999x coverage. Some samples contained two strains, the strain with the highest depth of coverage is denoted with “major” and the lowest with “minor” (e.g. “R01-04-major/minor”).

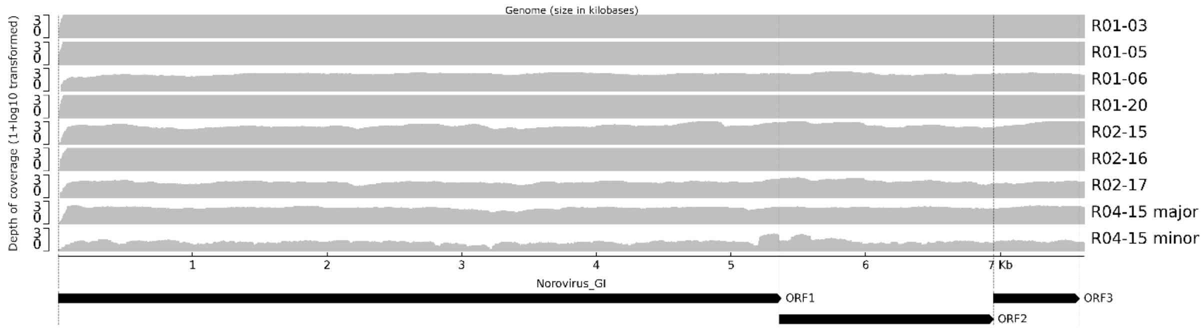

Supplemental Figure S2 – Sequence coverage plots of norovirus GI. Open Reading Frames (ORFs) are shown below the chart. The coverage is shown as a 1+log10 transformation with a maximum depth of 999x coverage. Sample R04-15 contained two strains, the strain with the highest depth of coverage is denoted with “major” and the lowest with “minor” (e.g. “R04-15-major/minor”).

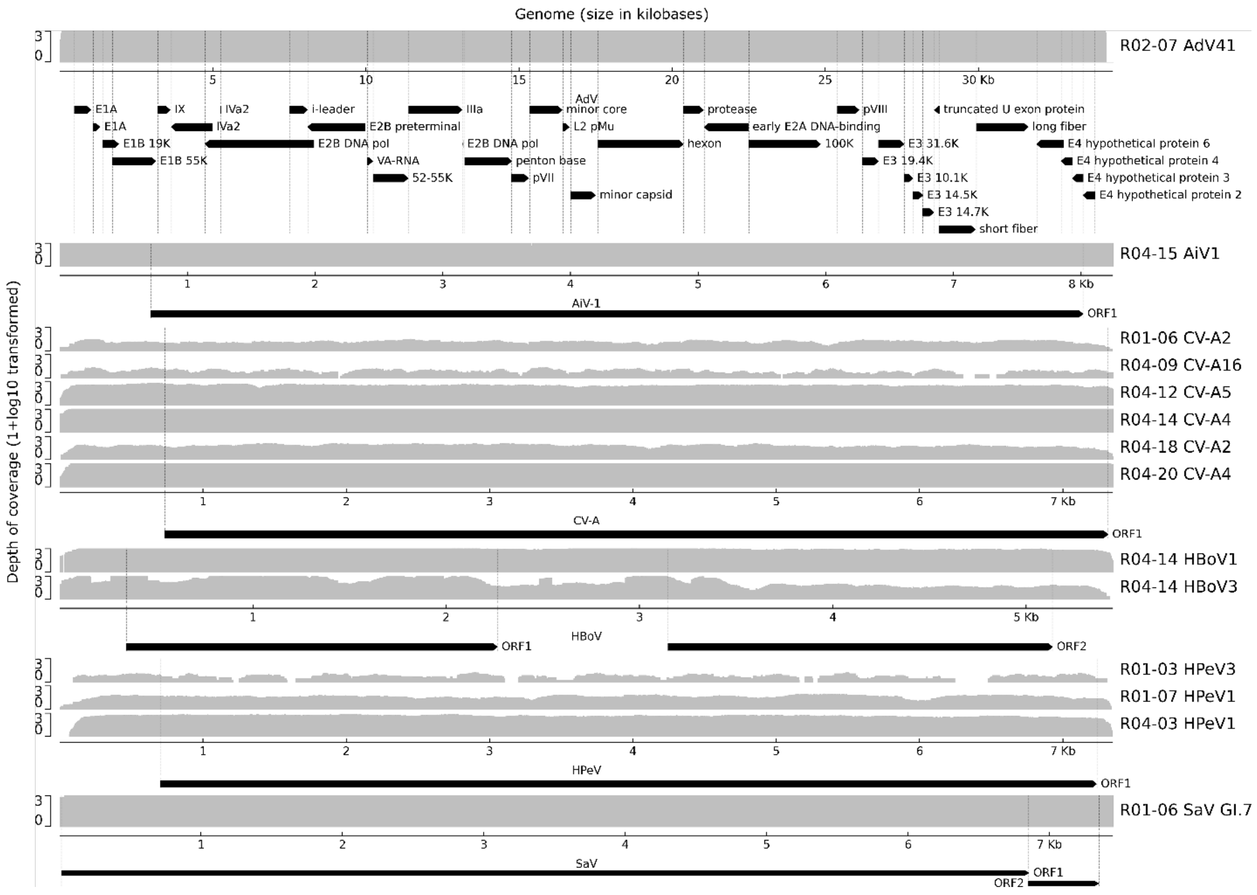

Supplemental Figure S3 – Sequence coverage plots of Adenovirus 41 (AdV41), Aichivirus (AiV), Coxsackievirus (CV), Human Bocaparvovirus (HBoV), Human Parechovirus (HPeV) and Sapovirus (SaV) GI.7. Open Reading Frames (ORFs) are shown below the chart. The coverage is shown as a 1+log10 transformation with a maximum depth of 999x coverage.

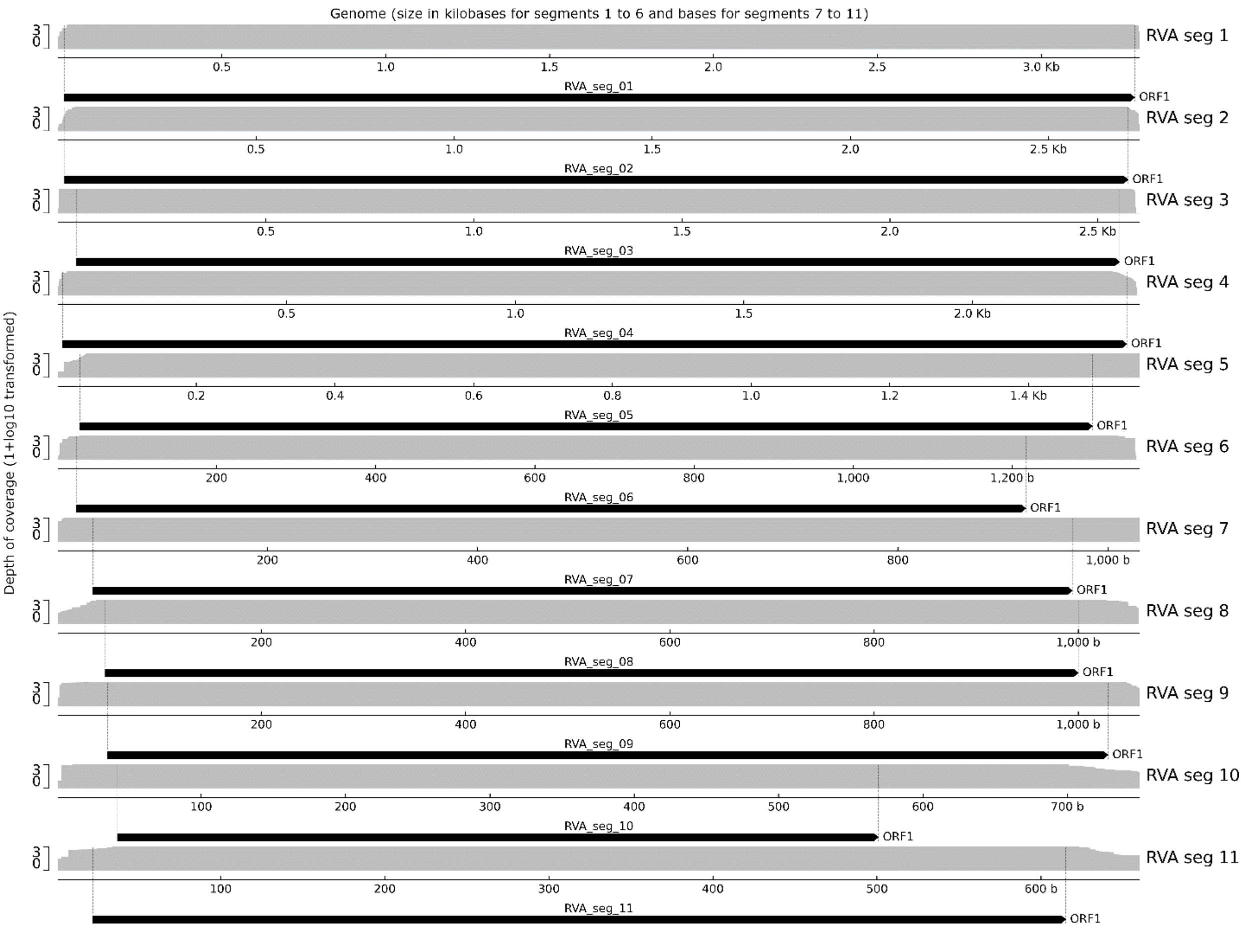

Supplemental Figure S4 – Sequence coverage plots of Rotavirus A (RVA). Open Reading Frames (ORFs) are shown below the chart. The coverage is shown as a 1+log10 transformation with a maximum depth of 999x coverage.

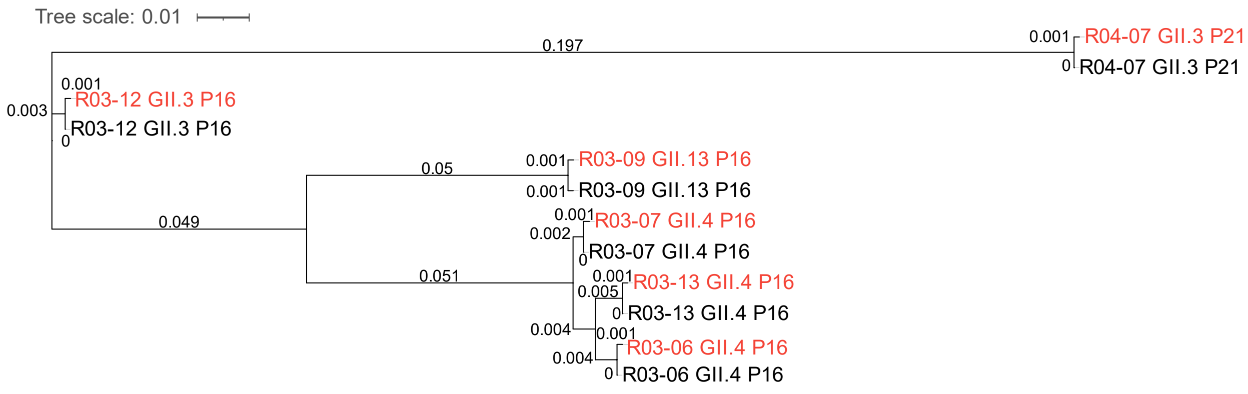

Supplemental Figure S5 – Maximum likelihood tree of the 969nt ORF1/2 region of samples with discordant WGS (red) and Sanger sequences. Five sample had discordant WGS and Sanger sequences: R03-06, R03-13, R03-07, R04-07 had 1 SNP pairwise distance, R03-09 had 2 SNP pairwise distance. To generate the tree a GTR substitution model was used. The scale bar represents nucleotide substitutions per site and the labels show branch lengths.

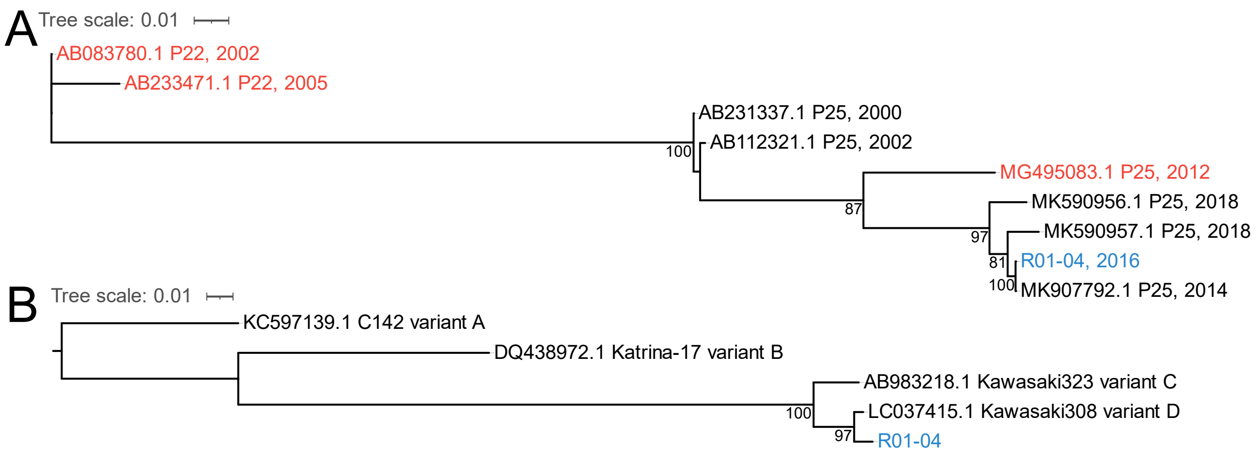

Supplemental figure S6 – Phylogenetic analysis of norovirus GII.17[P25].

Sequences of the GII.17[P25] strain from sample R01-04 are depicted in blue. A) The polymerase tree is based on the 783nt long 3'-end of ORF1 (RdRp) with P22 as the outgroup, alongside several public P25 sequences labeled with their sampling year. Reference sequences from Chhabra et al., 2019, are shown in red. The tree was inferred with the TNe model. B) The maximum likelihood VP1 tree is based on the 1633nt long ORF2 of GII.17 variants A-D (Parra et al., 2017), with the ancestral GII.17 variant A as the outgroup. The tree was inferred with the TIM2e model. The scale bar represents nucleotide substitutions per site and selected bootstrap values >70 are shown.
